# Factors associated with ultra-processed foods consumption in a cohort of Brazilian pregnant women

**DOI:** 10.1590/0102-311XEN177022

**Published:** 2023-06-26

**Authors:** Ana Claudia Santos Amaral Fraga, Mariza Miranda Theme, Maria Pappaterra Bastos

**Affiliations:** 1 Instituto Nacional de Infectologia Evandro Chagas, Fundação Oswaldo Cruz, Rio de Janeiro, Brasil.; 2 Escola Nacional de Saúde Pública Sergio Arouca, Fundação Oswaldo Cruz, Rio de Janeiro, Brasil.; 3 Departamento de Informática do SUS, Rio de Janeiro, Brasil.

**Keywords:** Ultra-processed Foods, Pregnancy, Maternal Nutrition, Cohort Studies, Alimentos Ultraprocessados, Gravidez, Nutrição Materna, Estudos de Coortes, Alimentos Ultraprocesados, Embarazo, Nutrición Materna, Estudios de Cohortes

## Abstract

Nutrition during pregnancy is essential for the health of the pregnant woman, the development of the fetus, and the prevention of complications related to pregnancy and the postpartum period. This study described the factors associated with high consumption of ultra-processed foods among pregnant women. This prospective cohort study was performed from February 2016 to November 2019 in two health units in the city of Rio de Janeiro, Brazil, with data from 344 pregnant women. The first interview was conducted in the prenatal visit at less than 20 gestational weeks, the second at 34 gestational weeks, and the third at two months postpartum. Diet was assessed in the last interview using a food frequency questionnaire, and food items were classified according to NOVA. The percentage of ultra-processed foods consumption was estimated by tertile distribution, and the third tertile represented the highest consumption. Based on the hierarchical analysis model, the associations between ultra-processed foods consumption and sociodemographic, reproductive health, pregestational, behavioral, and pregnancy variables were assessed using a multinomial logistic regression model. Older women had lower ultra-processed foods consumption (OR = 0.33; 95%CI: 0.15-0.71). Few years of schooling (up to 7 years; OR = 5.58; 95%CI: 1.62-19.23), history of a previous childbirth (OR = 2.48; 95%CI: 1.22-5.04), history of two or more previous childbirths (OR = 7.53; 95%CI: 3.02-18.76), and no history of regular physical activity before pregnancy (OR = 2.40; 95%CI: 1.31-4.38) were risk factors. The identification of risk and protection factors allows for the establishment of control measures and encouragement of healthy practices during prenatal care.

## Abstract (pt)

A nutrição durante a gravidez é essencial para a saúde da gestante, o desenvolvimento do bebê e a prevenção de complicações relacionadas à gravidez e ao pós-parto. Este estudo descreveu os fatores associados ao alto consumo de alimentos ultraprocessados entre gestantes. Trata-se de uma coorte prospectiva realizada de fevereiro de 2016 a novembro de 2019, em duas unidades de saúde do Município do Rio de Janeiro, Brasil, que analisou dados de 344 gestantes. A primeira entrevista foi realizada na consulta pré-natal com menos de 20 semanas de gestação, a segunda com 34 semanas de gestação e a terceira dois meses após o parto. A dieta foi avaliada na última entrevista por meio de um questionário de frequência alimentar e os itens alimentares foram classificados de acordo com a classificação NOVA. O percentual de consumo de alimentos ultraprocessados foi calculado em tercis de distribuição, dos quais o terceiro tercil representou o maior consumo. Com base no modelo de análise hierárquica, as associações entre o consumo de alimentos ultraprocessados e variáveis sociodemográficas, de saúde reprodutiva, pré-gestacionais, comportamentais e gestacionais foram investigadas usando um modelo de regressão logística multinomial. Mulheres mais velhas apresentaram menor consumo de alimentos ultraprocessados (OR = 0,33; IC95%: 0,15-0,71). Os fatores de risco foram baixa escolaridade (até sete anos; OR = 5,58; IC95%: 1,62-19,23), histórico de parto anterior (OR = 2,48; IC95%: 1,22-5,04), histórico de dois ou mais partos anteriores (OR = 7,53; IC95%: 3,02-18,76) e ausência de histórico de atividade física regular antes da gestação (OR = 2,40; IC95%: 1,31-4,38). A identificação de fatores de risco e proteção permite o estabelecimento de medidas de controle e o incentivo a práticas saudáveis durante o pré-natal.

## Abstract (es)

La nutrición durante el embarazo es esencial para la salud de la futura madre, el desarrollo del bebé y la prevención de complicaciones relacionadas con el embarazo y el posparto. Este estudio describió los factores asociados con el alto consumo de alimentos ultraprocesados entre las mujeres embarazadas. Se trata de una cohorte prospectiva realizada entre febrero de 2016 y noviembre de 2019, en dos unidades de salud de la ciudad de Río de Janeiro, Brasil, que analizó datos de 344 gestantes. La primera entrevista se realizó en la visita prenatal a las 20 semanas de gestación, la segunda a las 34 semanas de gestación y la tercera dos meses después del parto. La dieta se evaluó en la última entrevista mediante un cuestionario de frecuencia de alimentos y los alimentos se clasificaron de acuerdo con la clasificación NOVA. El porcentaje de consumo de alimentos ultraprocesados se calculó en terciles de distribución, de los cuales el tercer tercil representó el mayor consumo. Con base en el modelo de análisis jerárquico, se investigaron las asociaciones entre el consumo de alimentos ultraprocesados y las variables sociodemográficas, de salud reproductiva, previas al embarazo, conductuales y gestacionales mediante un modelo de regresión logística multinomial. Las mujeres mayores presentaron menor consumo de alimentos ultraprocesados (OR = 0,33; IC95%: 0,15-0,71). Los factores de riesgo fueron bajo nivel educativo (hasta siete años; OR = 5,58; IC95%: 1,62-19,23), antecedentes de parto previo (OR = 2,48; IC95%: 1,22-5,04), antecedentes de dos o más partos previos (OR = 7,53; IC95%: 3,02-18,76) y sin antecedentes de actividad física regular antes del embarazo (OR = 2,40; IC95%: 1,31-4,38). La identificación de factores de riesgo y protección permite el establecimiento de medidas de control y el fomento de prácticas saludables durante la atención prenatal.

## Introduction

Diet quality has changed over the years, with a reduction in the consumption of fruits, vegetables, grains, and legumes, and an increase in the consumption of industrially processed foods and beverages and ready-to-eat food products ^1^. Analyzing these changes in dietary patterns, Monteiro et al. ^2^ developed a food classification based on the level of processing and the nature, extent, and purpose of industrial processes to foods. The NOVA system is internationally recognized and has been widely used in epidemiological studies on individual food consumption, diet quality, and health conditions ^3^ ^,^ ^4^ ^,^ ^5^.

Ultra-processed foods are industrial formulations with little or no real food that are marketed for quick consumption ^6^ ^,^ ^7^. They have a negative effect on diet quality due to their high levels of sodium, saturated fat, and sugar ^8^, which are important factors related to morbidity and mortality from noncommunicable diseases ^9^. Recent studies in adults have shown an association between high ultra-processed foods consumption and an increased risk of overweight/obesity, cancer, type 2 diabetes, cardiovascular diseases, and all-cause mortality ^3^ ^,^ ^4^ ^,^ ^10^.

Data from the literature have consistently shown that, during pregnancy, a diet based on healthy eating habits contributes to the health of the pregnant woman, the development of the fetus, and the prevention of complications related to the pregnancy and the postpartum period ^11^ ^,^ ^12^ ^,^ ^13^. Recently, studies on ultra-processed foods observed an important relationship between high consumption and increased gestational weight gain, gestational diabetes, overweight/obesity, and depression and sadness ^7^ ^,^ ^14^ ^,^ ^15^. Inadequate maternal weight gain favors the development of gestational and postpartum complications, besides influencing fetal health conditions, such as birth weight, mode of delivery, and duration of pregnancy ^16^.

Sociodemographic, cultural, and behavioral factors can compromise maternal eating habits and thus lead to increased ultra-processed foods consumption. Identifying these factors, especially potentially modifiable factors, can support more effective nutritional guidance measures. Given the increase in ultra-processed foods consumption in medium-/low-income countries, this study aims to describe the factors associated with higher ultra-processed foods consumption, based on data from a cohort of pregnant women in two Family Health Strategy (FHS) health units in the city of Rio de Janeiro, Brazil.

## Methodology

### Study design and population

This study analyzed data from pregnant women who participated in the research project entitled *Factors Associated with Pregestational Obesity and its Repercussions on Maternal and Neonatal Health*, a prospective cohort study conducted from February 2016 to November 2019 in two FHS units. The health units are located in one of the most vulnerable regions of Rio de Janeiro city, with the fifth lowest human development index in the city ^17^.

This sample size was estimated for a 5% prevalence of negative outcomes (gestational diabetes or hypertension), 95% confidence interval (95%CI), and 80% power, allowing the detection of a difference of ≥ 2 in relative risk, considering a ratio of about 3:1 (35% overweight/obesity) between exposed and unexposed. In total, 512 pregnant women with a low obstetric risk, gestational age < 20 weeks, and aged ≥ 18 years were included in the baseline study. The first interview was performed during prenatal visits, where pregnant women were recruited sequentially until the planned sample size was reached. Two more interviews were conducted: at 34 weeks gestational age and two months postpartum. Women who answered the three questionnaires were included in this analysis, totaling 393 women.

### Outcome variable: consumption of ultra-processed foods

Food consumption during pregnancy was assessed using a food frequency questionnaire (FFQ) applied in the third interview. The questionnaire presented eight different options for consumption frequency that were converted into daily intake: “more than three times per day”, “two to three times per day”, “once per day”, “five to six times per week”, “two to four times per week”, “once per week”, “one to three times per month”, and “never/almost never”. The list of foods included 88 items and, for each item, standardized portions, as an option to assess the amount consumed ^18^. The questionnaire was validated by Giacomello et al. ^19^ among pregnant women who used public healthcare services in Brazil.

Food energy value was estimated by converting the daily intake, consulting a food consumption table and giving references in 100-g portions and household measurements ^20^ ^,^ ^21^. ultra-processed foods were identified according to the NOVA classification proposed in the *Dietary Guidelines for the Brazilian Population* ^22^, which considers the following food groups: (1) natural or minimally processed foods; (2) oils, fats, salt, and sugar; (3) processed foods; and (4) ultra-processed foods. The variable ultra-processed foods did not follow a Gaussian distribution and, therefore, was analyzed by tertile distribution. The third tertile corresponded to the highest consumption and the first tertile was the reference category in the analysis with the second and third tertiles.

### Covariables

First questionnaire: age (18-24; 25-34; ≥ 35 years old); years of schooling (< 7; 8-11; ≥ 12 years); ethnicity/skin-color (white, black, and mixed-race); paid work (“Do you currently have a job that you earn money with?”: yes/no); marital status (“Do you live with a spouse/partner?”: yes/no); parity (no previous childbirths; one previous childbirth; two or more previous childbirths); planned pregnancy (yes, if the woman “wanted to become pregnant” versus no, if the woman “wanted to wait longer” or “did not want to become pregnant”); satisfaction with weight before pregnancy (yes/no); leisure time physical activity before pregnancy based on women’s information (yes/no); social support (high; above the median on the scale developed in the *Medical Outcomes Study*) ^23^.

Second questionnaire: smoking during pregnancy (at least one cigarette per day every day); alcohol abuse (2 on the TWEAK scale) ^24^; diabetes mellitus (diagnosis of gestational or pregestational diabetes); hypertension (diagnosis of gestational or pregestational hypertension); prenatal nutritional guidance (yes/no).

Third questionnaire: pregestational nutritional status (classified according to the body mass index [BMI], measured until the 13th gestational week and recorded in the woman’s prenatal booklet); leisure time physical activity during pregnancy (according to the *Pregnancy Physical Activity Questionnaire* [PPAQ] ^25^, pregnant women are classified as active [≥ 150 minutes/week] or insufficiently active or inactive [< 150 minutes/week]); symptoms of depression (≥ 10 on the *Edinburgh Postnatal Depression Scale* [EPDS]) ^26^; symptoms of anxiety (≥ 3 on the *Patient Health Questionnaire-4* [PHQ4]).

### Data analysis

To assess food consumption during pregnancy, a sectional analysis of the FFQ in the third wave was performed. Foods were initially quantified according to their energy value and percentage contribution to total daily energy intake, grouped according to the NOVA classification.

(1) Natural or minimally processed food: rice; pasta; beans and legumes (lentil); fruits (orange, banana, papaya, apple, watermelon, pineapple, mango, grape, pear, passion fruit, lemon, watermelon, avocado, and guava); root and tuber vegetables (potato, cassava, carrot, and beet); milk, chicken; red meat; juice; fish; eggs; giblets; flour (cassava or manioc flour, and polenta); peanuts; popcorn; and coffee.

(2) Oils, fats, salt, and sugar: sugar; butter.

(3) Processed foods: cheese; canned foods (maize, peas, and tuna/sardine); bacon.

(4) Ultra-processed foods: bread; cookies; soft drinks; cakes; chocolate bars; pizza; candies; caramels; ice cream; chocolate powder; margarine; mayonnaise; yogurt; processed meats (hamburger and sausage) and alcoholic beverages (beer and wine).

**Figure 1:**
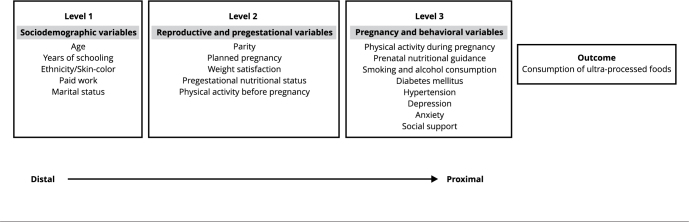
Theoretical model of consumption of ultra-processed foods during pregnancy.

The analysis excluded women with implausible total energy intake (< 600 or > 6,000Kcal/day) ^27^. A bivariate analysis of the independent variables in relation to ultra-processed foods consumption was performed using the chi-square test and 5% statistical significance. Factors associated with ultra-processed foods consumption were identified based on the literature on the topic among pregnant women, introducing a set of covariables into the analysis using a hierarchical multinomial logistic regression model. The underlying logic of the proposed model is that the hierarchically superior factors use the inferior factors to perform their actions (Figure 1).

Variables at level 1 that reached significance (p < 0.20) in the simple logistic regression remained in the multivariate regression model, adopting the backward procedure with gradual exclusion of the variables with the lowest statistical significance, until the final model at this level retained only variables with p < 0.05.

For each variable at level 2, the adjusted odds ratio (OR) was estimated for the variables retained at the level 1. Variables that reached statistical significance (p < 0.20) were included in the multivariate regression model at this level, along with the variables retained at the previous level. A new backward procedure with gradual exclusion of the variables with the lowest statistical significance was performed until the model retained only variables with p < 0.05. Finally, the same procedures were performed at level 3.

The final model for the hierarchical multivariate logistic regression analysis retained only variables with p < 0.05 at each level. Statistical analyses were performed using SPSS version 22 (https://www.ibm.com/).

### Ethical aspects

The study was approved according to the recommendations of *Resolution n. 466/2012* of the Brazilian National Health Council, which defines the procedures for research in human subjects, and has been filed with the Ethics Research Committee of the Sergio Arouca National School of Public Health, Oswaldo Cruz Foundation (ENSP/Fiocruz, CAAE 21982613.6.0000.5240).

## Results

Of the 520 pregnant women recruited for the first interview, 393 completed the three questionnaires. Losses (n = 120; 23.4%) occurred mainly due to address changes (n = 63; 12.3%), refusal to participate (n = 25; 4.9%), miscarriage or stillbirth (n = 17, 3.3%), and not located (n = 15; 2.9%). Years of schooling (p = 0.011) was the only variable with a significant difference between respondents and nonrespondents. We found no significant difference in age (p = 0.272), ethnicity/skin-color (p = 0.650), paid work (p = 0.388), or marital status (p = 0.958) (Table 1).

**Table 1:** Comparative analysis of sociodemographic characteristics between respondents and nonrespondents in a cohort study with pregnant women. Rio de Janeiro, Brazil, 2016-2019.

Characteristics	Total	Respondents	Nonrespondents	p-value
	n (%)	n (%)	n (%)	
Age (years)				0.272
18-24	233 (45.4)	173 (44.0)	60 (50.0)	
25-29	140 (27.3)	106 (27.0)	34 (28.3)	
≥ 30	140 (27.3)	114 (29.0)	26 (21.7)	
Years of schooling				0.011 *
Up to 7	211 (41.1)	156 (39.7)	55 (45.8)	
8-11	260 (50.7)	211 (53.7)	49 (40.8)	
≥ 12	41 (8.0)	26 (6.6)	15 (12.5)	
Ethnicity/skin-color				0.650
White	144 (29.0)	112 (29.5)	32 (27.4)	
Mixed-race	245 (49.3)	189 (49.7)	56 (47.9)	
Black	108 (21.7)	79 (20.8)	29 (24.8)	
Marital status				0.794
Lives with a spouse/partner	397 (77.5)	305 (77.8)	92 (76.7)	
Does not live with a spouse/partner	115 (22.5)	87 (22.2)	28 (23.3)	
Paid work				0.388
Yes	228 (44.6)	179 (45.7)	49 (41.2)	
No	283 (55.4)	213 (54.3)	70 (58.8)	

* Chi-square test, p < 0.05.

After excluding implausible total energy intake (< 600 or > 6,000Kcal/day), we analyzed data from 344 pregnant women. Mean daily energy intake during pregnancy was 3,335Kcal (standard deviation - SD ±1,147.57Kcal), of which 52.5% (1,752.2Kcal) came from unprocessed or minimally processed foods, 4.8% (161.4Kcal) from cooking ingredients, 7.3% (242.7Kcal) from processed foods, and 35.3% (1,178.6Kcal) from ultra-processed foods (Table 2). The group 1 foods that contributed the most were fruits (8.4%), beans and legumes (7.8%), root and tuber vegetables (5.9%), rice (5.6%), milk (5%), chicken (4%), red meat (3.9%), juice (2.2%), fish (1.8%), pasta (1.7%), and eggs (1.4%). In the cooking ingredients group, sugar and butter contributed 4.5% and 0.4%, respectively. In group 3, cheese (1%), canned foods (0.5%), and bacon (0.3%) contributed the highest percentage of calories. The most widely consumed ultra-processed foods were breads (9.8%), cookies (5.8%), soft drinks (3.2%), crackers (2.1%), cakes (2%), chocolate bars (1.5%), pizza (1.4%), processed meat (1.3%), ice cream (1.1%), and chocolate powder (1%).

**Table 2:** Percentage of energy from foods grouped according to the NOVA classification during pregnancy (n = 344). Rio de Janeiro, Brazil, 2016-2019.

Food groups	Consumption during pregnancy	
	Kcal/day	%
Group 1	1,752.2	52.5
Fruits	279.9	8.4
Beans and legumes	261.2	7.8
Root and tuber vegetables	195.7	5.9
Rice	188.5	5.6
Milk	167.1	5.0
Chicken	132.9	4.0
Red meat	131.4	3.9
Juice	72.0	2.2
Fish	58.6	1.8
Pasta	56.7	1.7
Eggs	47.7	1.4
Group 2	161.4	4.8
Sugar	149.5	4.5
Butter	11.9	0.4
Group 3	242.7	7.3
Cheese	34.1	1.0
Canned foods (maize, peas, and tuna/sardine)	17.8	0.5
Bacon	11.6	0.3
Group 4	1,178.6	35.3
Bread	327.5	9.8
Cookies	194.0	5.8
Soft drinks	108.0	3.2
Crackers	71.0	2.1
Cakes	67.3	2.0
Chocolate bars	49.0	1.5
Pizza	45.3	1.4
Processed meat	42.3	1.3
Ice cream	35.7	1.1
Chocolate powder	34.5	1.0

Note: Group 1: natural or minimally processed foods; Group 2: oils, fats, salt, and sugar; Group 3: processed foods; Group 4: ultra-processed foods.

The mean age of respondents was 26.7 years (SD ±6.0), ranging from 18 to 44 years. They reported a mean of 10.1 (SD ±2.8) years of schooling and 56.4% had eight to eleven years of schooling. Table 3 shows the characteristics of pregnant women according to their energy intake from ultra-processed foods. Compared with the 1st tertile of consumption (lowest consumption), women in the 3rd tertile (highest consumption) were younger, had fewer years of schooling, reported less physical activity before pregnancy, and were more likely to report alcohol abuse. Obstetric variables, pregestational nutritional status, and psychological variables, such as depression and anxiety during pregnancy, did not show statistically different proportions between tertiles of ultra-processed foods consumption.

**Table 3:** Characteristics of pregnant women according to energy intake from ultra-processed foods during pregnancy (n = 344). Rio de Janeiro, Brazil, 2016-2019.

Hierarchical level and variables	Consumption of ultra-processed foods			
	n (%)	1st tertile (%)	2nd tertile (%)	3rd tertile (%)	p-value
Level 1					
Age (years)					< 0.001
18-24	147 (42.7)	23.8	29.3	46.9	
25-29	92 (26.7)	31.5	39.1	29.3	
≥ 30	105 (30.5)	48.6	34.3	17.1	
Mean age (SD)	26.7 (6.0)	28.9 (6.3)	26.7 (6.0)	24.5 (4.8)	< 0.001
Years of schooling					< 0.001
Up to 7	126 (36.6)	25.4	31.0	43.7	
8-11	194 (56.4)	35.6	36.1	28.4	
≥ 12	24 (7.0)	58.3	25.0	16.7	
Mean years of schooling (SD)	10.1 (2.8)	10.7 (2.8)	10.0 (2.7)	9.8 (2.8)	0.05
Ethnicity/Skin-color					0.24
White	102 (29.7)	37.3	36.3	26.5	
Mixed-race	165 (48.0)	32.1	33.9	33.9	
Black	64 (18.6)	29.7	26.6	43.8	
Paid work					0.49
Yes	157 (45.6)	35.0	35.0	29.9	
No	186 (54.1)	31.7	32.3	36.0	
Marital status					0.68
Lives with a spouse/partner	268 (77.9)	34.2	32.1	33.6	
Does not live with a spouse/partner	75 (21.8)	30.7	37.3	32.0	
Level 2					
Parity					0.17
No previous childbirths	118 (34.3)	39.8	27.1	33.1	
1 previous childbirth	146 (42.4)	32.9	37.0	30.1	
≥ 2 previous childbirths	80 (23.3)	25.0	36.3	38.8	
Planned pregnancy					0.09
Wanted to become pregnant	159 (46.2)	39.0	32.7	28.3	
Did not want to become pregnant	185 (53.8)	28.6	34.1	37.3	
Satisfaction with weight before pregnancy					0.71
Yes	202 (58.7)	31.7	34.2	34.2	
No	142 (41.3)	35.9	32.4	31.7	
Pregestational nutritional status					0.23
Underweight	16 (4.7)	18.8	18.8	62.5	
Adequate	131 (38.1)	31.3	35.1	33.6	
Overweight	86 (25.0)	36.0	36.0	27.9	
Obesity	110 (32.0)	36.4	30.9	32.7	
Leisure time physical activity before pregnancy					< 0.001
Yes	120 (34.9)	45.0	31.7	23.3	
No	223 (64.8)	27.4	34.1	38.6	
Level 3					
Leisure time physical activity during pregnancy					0.13
Inactive/Insufficiently active	311 (90.4)	31.8	33.8	34.4	
Active	33 (9.6)	48.5	30.3	21.2	
Nutritional guidance during pregnancy					0.77
Yes	282 (82.0)	34.0	33.3	32.6	
No	39 (11.3)	28.2	35.9	35.9	
Smoking during pregnancy					0.29
Yes	25 (7.3)	20.0	36.0	44.0	
No	319 (92.7)	34.5	33.2	32.3	
Alcohol use during pregnancy					0.07
Does not drink/Drinks without abuse	258 (75.0)	36.8	31.4	31.8	
Alcohol abuse	86 (25.0)	23.3	39.5	37.2	
Diagnosis of diabetes (pre- or gestational)					0.27
Yes	30 (8.7)	46.7	26.7	26.7	
No	308 (89.5)	32.1	34.7	33.1	
Diagnosis of hypertension (pre- or gestational)					0.15
Yes	55 (16.0)	32.7	43.6	23.6	
No	289 (84.0)	33.6	31.5	34.9	
Symptoms of depression during pregnancy					0.20
Yes	117 (34.0)	27.4	35.0	37.6	
No	227 (66.0)	36.6	32.6	30.8	
Anxiety during pregnancy					0.13
Yes	83 (24.1)	25.3	41.0	33.7	
No	261 (75.9)	36.0	31.0	33.0	
Social support					0.85
High	172 (50.0)	34.9	32.6	32.6	
Low	172 (50.0)	32.0	34.3	33.7	

SD: standard deviation.

Tables 4 and 5 present the results of the analysis of the three hierarchical levels, comparing the 2nd and 3rd tertiles with the 1st tertile of ultra-processed foods consumption (reference). In the comparative analysis between the 2nd with the 1st tertiles, two variables at level 1 (age and years of schooling) showed a crude association with ultra-processed foods consumption (Table 4). However, after multivariate analysis, no variable remained in the final model with a significance level < 0.05 (Table 5). At level 2, only parity and leisure time physical activity before pregnancy were associated with the 2nd tertile of ultra-processed foods consumption with a significance level < 0.20 (Table 4). After adjusting for the significant variables at this level and the previous level, only parity remained in the final model (p < 0.05) (Table 5). At level 3, the variables associated with the second tertile of ultra-processed foods consumption (p < 0.20) were alcohol abuse, diabetes mellitus, and anxiety disorder during pregnancy. All variables lost significance in the multivariate model, therefore, we did not include them in the final model.

**Table 4:** Crude analysis of variables of the three hierarchical levels and tertiles of consumption of ultra-processed foods during pregnancy. Rio de Janeiro, Brazil, 2016-2019.

Hierarchical level and variables	2nd tertile		3rd tertile	
	OR *	95%CI	OR **	95%CI
Level 1				
Age (years)				
18-24	0.99	0.51-1.92	2.12	1.09-4.11 ***
25-29	1.00	-	1.00	-
≥ 30	0.57	0.30-1.09 ***	0.38	0.18-0.60
Years of schooling				
Up to 7	2.84	0.98-8.24 ***	6.02	1.82-19.84 ***
8-11	2.37	0.86-6.52 ***	2.79	0.87-8.96 ***
≥ 12	1.00	-	1.00	-
Ethnicity/Skin color				
White	1.00	-	1.00	-
Mixed-race	1.08	0.60-1.95	1.49	0.80-2.76
Black	0.92	0.41-2.04	2.07	0.97-4.45
Paid work				
Yes	1.00	-	1.00	-
No	1.02	0.61-1.71	1.33	0.79-2.24
Marital status				
Lives with a spouse/partner	1.00	-	1.00	-
Does not live with a spouse/partner	1.30	0.70-2.43	1.07	0.56-2.03
Level 2				
Parity				
No previous childbirths	1.00	-	1.00	-
1 previous childbirth	2.54	1.27-5.09	2.19	1.07-4.48 ***
≥ 2 previous childbirths	4.17	1.65-10.52	5.75	2.18-15.14 ***
Planned pregnancy				
Wanted to become pregnant	1.00	-	1.00	-
Did not want to become pregnant	1.35	0.79-2.30	1.44	0.81-2.52
Satisfaction with weight before pregnancy				
Yes	1.00	-	1.00	-
No	0.84	0.49-1.45	1.03	0.580-1.816
Pregestational nutritional status				
Underweight	0.84	0.16-4.53	2.63	0.62-11.11 ***
Adequate	1.00	-	1.00	-
Overweight	0.92	0.47-1.79	0.85	0.41-1.76
Obesity	0.80	0.42-1.51	1.08	0.55-2.10
Leisure time physical activity before pregnancy				
Yes	1.00	-	1.00	-
No	1.70	0.98-2.94 ***	2.27	1.24-4.13 ***
Level 3				
Leisure time physical activity during pregnancy				
Active	1.00	-	1.00	-
Inactive/Insufficiently active	1.09	0.45-2.65	1.22	0.44-3.37
Nutritional guidance during pregnancy				
Yes	1.00	-	1.00	-
No	1.60	0.64-3.86	1.69	0.66-4.36
Smoking during pregnancy				
Yes	1.41	0.44-4.52	1.62	0.51-5.19
No	1.00	-	1.00	-
Alcohol use during pregnancy				
Alcohol abuse	1.69	0.81-3.25 ***	1.43	0.72-2.84
Does not drink/Drinks without abuse	1.00	-		
Diagnosis of diabetes (pre- or gestational)				
Yes	0.46	0.18-1.22 ***	0.55	0.20-1.52
No	1.00	-		
Diagnosis of hypertension (pre- or gestational)				
Yes	1.34	0.65-2.80	0.65	0.27-1.56
No	1.00	-		
Symptoms of depression during pregnancy				
Yes	1.40	0.77-2.55	1.57	0.84-2.93 ***
No	1.00	-		
Anxiety during pregnancy				
Yes	1.90	1.00-3.61 ***	1.53	0.77-3.07
No	1.00	-		
Social support				
Yes	1.00	-		
No	0.94	0.55-1.63	0.80	0.45-1.43

95%CI: 95% confidence interval; OR: odds ratio.

Note: reference category: 1st tertile.

* OR of the variables at level 2 in the hierarchical model that were adjusted for the variables retained at level 1 (age and years of schooling);

** OR of the variables at level 3 in the hierarchical model that were adjusted for the variables retained at levels 1 (age) and 2 (parity and leisure time physical activity before pregnancy);

*** Independent variables associated with the outcome with significance level p < 0.20, showing that the variable was included in the multivariate analysis at its hierarchical level.

**Table 5:** Final hierarchical model for the multivariate logistic regression analysis of the relationship between independent variables and tertiles of consumption of ultra-processed foods during pregnancy. Rio de Janeiro, Brazil, 2016-2019.

Hierarchical level and variables	2nd tertile		3rd tertile	
	aOR *	95%CI	aOR *	95%CI
Level 1				
Age (years)				
18-24	0.92	0.47-1.80	1.85	0.94-3.66
25-29	1.00	-	1.00	-
≥ 30	0.54	0.28-1.04	0.33	0.15-0.71
Years of schooling				
Up to 7	2.96	1.00-8.70	5.58	1.62-19.23
8-11	2.33	0.84-6.47	2.43	0.73-8.09
≥ 12	1.00	-	1.00	-
Level 2				
Parity				
No previous childbirths	1.00	-	1.00	-
1 previous childbirth	2.49	1.26-4.94	2.48	1.22-5.04
≥ 2 previous childbirths	4.11	1.72-9.80	7.53	3.02-18.70
Leisure time physical activity before pregnancy				
Yes	1.00	-	1.00	-
No	1.62	0.94-2.81	2.40	1.31-4.38

95%CI: 95% confidence interval; aOR: adjusted odds ratio.

Note: reference category: 1st tertile.

* Effect of each variable adjusted for the variables at the same hierarchical level that remained with p < 0.05 at the end of the multivariate analysis, and for the variables retained at the previous levels, referring to the strength of the associations adjusted at the entry level for each of these variables in the hierarchical model.

When comparing the 3rd tertile with the reference category, the variables at level 1 that showed an association with p < 0.20 were age, years of schooling, and ethnicity/skin-color (Table 4). In the multivariate model, the variable ethnicity/skin-color lost statistical significance (Table 5). At level 2, for parity, leisure time physical activity before pregnancy, and pregestational weight, p < 0.20 (Table 4). The only variables that remained in the final model were parity and leisure time physical activity before pregnancy (Table 5). No variable at level 3 showed statistical significance among women in the highest tertile of consumption, either in the analysis adjusted for variables retained at levels 1 and 2 or in the multivariate model among variables at the same level (Table 4). Thus, no variables at level 3 were included in the final model for high ultra-processed foods consumption among pregnant women in this cohort study (Table 5).

The final hierarchical model (Table 5) included the variables age and years of schooling (level 1), and parity and regular leisure time physical activity before pregnancy (level 2). Pregnant women with up to seven years of schooling were more than five times more likely to belong to the 3rd tertile of ultra-processed foods consumption (OR = 5.58; 95%CI: 1.62-19.23) compared with pregnant women with 12 or more years of schooling (reference). Pregnant women with two or more previous childbirths were more than four times (OR = 4.11; 95%CI: 1.72-9.80) more likely to belong to the 2nd tertile of consumption and seven times (OR = 7.53; 95%CI: 3.02-18.70) more likely to belong to the 3rd tertile compared with pregnant women with no previous childbirth (reference). Moreover, the lack of leisure time physical activity before pregnancy increased by twice the odds of high ultra-processed foods consumption (OR = 2.40; 95%CI: 1.31-4.38). Meanwhile, women aged ≥ 30 years showed lower odds of high consumption of these foods (OR = 0.33; 95%CI: 0.15-0.71).

## Discussion

This study identified a set of factors associated with high ultra-processed foods consumption. Pregnant women with fewer years of schooling, higher parity, and no regular physical activity before pregnancy reported higher ultra-processed foods consumption. Moreover, our data showed a protective effect of age, as older pregnant women were less likely to consume ultra-processed foods.

According to Brazilian ^27^ ^,^ ^28^ and international studies ^29^, age seems to has an important effect on eating behavior. Unhealthy eating habits, including replacing regular meals with snacks, eating while watching TV, and consuming high energy-dense beverages, are behaviors related to younger individuals ^2^, who tend to be more susceptible to marketing appeals ^30^. On the other hand, older pregnant women tend to adhere to a “healthy awareness” pattern consisting mainly of a higher consumption of whole wheat bread, fruits, vegetables, skim milk, and white meat, among other healthy foods ^31^.

As in the general population, women’s diet and lifestyle before and during pregnancy are strongly influenced by their sociodemographic characteristics. Evidence consistently suggests a social gradient by which older women with more years of schooling and higher income, or other markers of wealth, adopt a “healthier” dietary pattern, scoring higher on nutritional quality scales ^32^.

A study on pregestational food consumption in a cohort of 454 Brazilian pregnant women found an independent association between dietary pattern and age and years of schooling. Women who adhered to “lentils, whole grains, and soups” dietary patterns were older and had more schooling than women with low adherence to this pattern. Women who adhered more to “snacks, sandwiches, sweets, and soft drinks” dietary patterns were younger and had less schooling ^33^. Similarly, a cohort of 5,664 pregnant women in New Zealand showed that the “junk food” pattern was positively associated with younger maternal age and fewer years of schooling. Moreover, pregnant women adhered less to the Ministry of Health’s Food and Nutrition Guidelines ^29^.

Another important point of this study was regular physical activity before pregnancy. Women classified as sedentary before becoming pregnant, according to the new World Health Organization (WHO) guidelines ^34^, reported higher ultra-processed foods consumption. Regular physical activity before pregnancy is a strong predictor of physical activity during pregnancy, and its benefits are widely reported in the literature ^35^. However, common problems in pregnancy, such as nausea, pain, and fatigue, may also interfere with women’s adherence to physical activity, contributing to a combination of unhealthy habits that pose potential risks of negative pregnancy outcomes ^36^. Health-related behavioral changes involve great complexity and, to be successful, they require involvement, motivation, and support. Thus, during prenatal care, it is important to understand and address the barriers involved in this process with nutritional information and encouragement of healthy habits, such as physical activity. Studies suggest that diet during pregnancy tends to reflect other health-related behaviors before and during pregnancy ^37^.

Parity is the most frequently assessed obstetric variable in studies on dietary patterns. Our results show a direct association with ultra-processed foods consumption in the adjusted model. However, this association has shown conflicting results in the literature, sometimes with positive, sometimes with negative effects. A systematic review of dietary patterns and diet quality in pregnant women confirmed this finding. Among the 10 studies that addressed this variable, five found an inverse association between parity and healthy eating; in four studies, the association was positive, and one found no association ^32^.

Based on the *Dietary Guidelines for the Brazilian Population* ^22^, the diet should be based on a wide variety of unprocessed or minimally processed foods, cooking ingredients and processed foods should be used sparingly, and ultra-processed foods consumption should be avoided. The latter group is particularly critical, since ultra-processed foods contains high calories, low nutritional value, and additives in their composition ^22^. During pregnancy, ultra-processed foods consumption may jeopardize placental and fetal growth and development ^38^ and increase the risk of gestational diabetes, hypertensive syndromes, and gestational weight gain, compromising the health of fetuses and mothers in the medium and long term ^7^ ^,^ ^39^.

The proportions of calories from ultra-processed foods and unprocessed or minimally processed foods were similar to the proportions found in other Brazilian studies with pregnant women, ranging from 48.8% to 55% for natural foods and 32% to 43% for ultra-processed foods ^27^ ^,^ ^40^. Some studies have shown slightly lower proportions of ultra-processed foods in the diet, ranging from 22.2% to 24.8% ^7^ ^,^ ^41^. However, the consumption of this food group has increased ^7^ ^,^ ^27^, with a large portion coming from bread, cookies, cold cuts, and soft drinks ^2^. Studies with population samples, especially in high-income countries, have shown that ultra-processed foods consumption represents more than half of the total daily energy intake ^42^ ^,^ ^43^, and this is also a reality among pregnant women ^44^. ultra-processed foods dominate the food supply in high-income countries and their consumption has increased rapidly in middle-income countries ^45^. The differences in the proportions of food groups between studies can be partly explained by the use of different dietary data collection tools, such as the 24-hour dietary recall, food frequency questionnaires, and even the classification of foods to differentiate the groups.

Although our study assessed a low-income population living in a social vulnerable area, our data showed, even in this scenario, differences in ultra-processed foods consumption. We found that less educated pregnant women were more likely to report higher ultra-processed foods consumption. The choices that constitutes the basis of healthier eating may be related to a lack of access to information and an understanding of the importance of good eating habits. Reinforcing guidance on the consumption of minimally processed foods, such as grains, legumes, fruits, greens and vegetables, unprocessed meat, and other foods, rather than ultra-processed products, such as sausages, cold cuts, and ready-to-eat dishes, can restore traditional cultural eating patterns.

Despite the methodological care in this study, the external validity of our results is limited to women with low socioeconomic status who receive prenatal and obstetric care in public healthcare services. Another key point is that dietary assessment is complex and involves recording and analyzing numerous foods and beverages consumed daily in varying amounts. The food frequency questionnaire is subject to recall bias and may underestimate or overestimate the consumption of certain food groups, thus influencing the resulting estimates. Moreover, the tool used in this study was developed in the 1990s and was not designed specifically to classify foods according to the degree of processing. Some foods were difficult to classify, since the food frequency questionnaire does not discriminate between homemade and ready-to-eat dishes. To maintain comparability with another Brazilian study on ultra-processed foods consumption during pregnancy, we used the same food grouping method ^27^.

Strengths of this study include the cohort design with follow-up of pregnant women from early pregnancy to the postpartum period. The hierarchical analysis model consisted of sociodemographic, obstetric, psychological, and health variables in pregnancy that are rarely addressed in observational studies on dietary patterns and diet quality, allowing both the evaluation of the crude effect of the variables and the control for confounding in the multivariate model, identifying the variables that best explained ultra-processed foods consumption.

The results suggest that potentially modifiable factors, such as physical activity (besides age, parity, and years of schooling), can guide nutritional guidance actions. The promotion of healthy eating practices even before pregnancy, with accessible guidance, including mainly minimally processed or unprocessed foods in the diet and a significant reduction in ultra-processed foods, will contribute to adequate gestational weight gain, an important indicator of pregnancy progress. During pregnancy, women and their families are more likely to follow guidelines that will benefit both the mother and the fetus. Studies on dietary interventions and encouragement of physical activity during pregnancy have shown a reduction in gestational weight gain and beneficial effects on women’s health ^46^. However, realistic policies and actions to control or reduce ultra-processed foods consumption should extended beyond education and information programs in health services. These policies and actions should focus on government programs for the entire society. In various health areas, such as tobacco and alcohol control, the combination of health education, public information campaigns, product labeling, and government guidelines has proven effective in reducing ultra-processed foods consumption, with a positive effect on noncommunicable diseases and population’s health ^47^.

Some strategies have been implemented aimed at raising awareness of healthy food consumption among the population, such as the publication of dietary guidelines and the application of warning labels. In particular, the *Dietary Guidelines for the Brazilian Population* ^22^, published in 2014, which introduced the NOVA classification, values the context of food consumption and the sociocultural importance of eating ^48^, clearly highlighting the principles and recommendations of adequate and healthy eating. This approach has been increasingly used for the classification of food groups in international studies in adults ^3^ ^,^ ^49^, pregnant women ^44^, and official Pan-American Health Organization (PAHO) reports ^50^.

The use of food warning labels is another important strategy. In Chile, one year after the implementation of this policy, a study showed that its participants (mothers of children aged two to 14 years with different socioeconomic levels) understood that labeling regulation had been implemented to combat childhood obesity in the country, causing changes in the eating habits of the Chilean population ^51^. In 2020, following in the footsteps of different Latin American countries, such as Uruguay, Peru, Ecuador, and Bolivia, Brazil passed new legislation on the nutritional labeling of packaged foods, aiming to clarify the nutritional information on food labels and help consumers make more conscious choices ^52^. These standards came into effect in October 2022. Thus, considering the multiple determinants of food practices and the complexity and challenges involved in shaping current food systems, these strategies aim to contribute to the promotion and fulfilment of the human right to adequate food.

## Conclusion

Sociodemographic factors are important risk factors associated with ultra-processed foods consumption in the general population, particularly in pregnant women. Moreover, leisure time physical activity before and during pregnancy is a potentially modifiable factor associated with lower consumption of these foods. An unhealthy diet before and during pregnancy can have negative consequences for the health of both mother and fetus. The identification of risk and protective factors allows for the establishment of control measures and the encouragement of healthy practices aimed at the most vulnerable population. However, the greatest benefits come from intervention strategies during prenatal care associated with public policies that reach the entire population.
